# Interculturality, public health and health education: data report based on the Virtual Learning Environment of the Brazilian Health System (AVASUS)

**DOI:** 10.3389/fpubh.2025.1642452

**Published:** 2025-09-01

**Authors:** Priscila Sanara da Cunha, Natalia Araújo do Nascimento Batista, Felipe Fernandes, Ingridy Barbalho, Manoel H. Romão, Karla M. D. Coutinho, Janaina Valentim, Juciano de Sousa Lacerda, Aline de P. Dias, Susana Henriques, Ricardo Valentim, Fátima Alves, Karilany D. Coutinho

**Affiliations:** ^1^Laboratory for Technological Innovation in Health (LAIS), Federal University of Rio Grande do Norte (UFRN), Natal, Brazil; ^2^Intercultural Relations Graduate Program, Universidade Aberta, Lisboa, Portugal; ^3^Health Sciences Research Unit: Nursing (UICISA: E), Nursing School of Coimbra (ESEnfC), Coimbra, Portugal; ^4^Center for Global Studies, Universidade Aberta, Lisboa, Portugal; ^5^Department of Social Communication, Federal University of Rio Grande do Norte, Natal, Brazil; ^6^Media Studies Graduate Program, Federal University of Rio Grande do Norte, Natal, Brazil; ^7^Health Management and Innovation Graduate Program, Federal University of Rio Grande do Norte, Natal, Brazil; ^8^Department of Educational Foundations and Policies, Federal University of Rio Grande do Norte, Natal, Brazil; ^9^Department of Biomedical Engineering, Federal University of Rio Grande do Norte, Natal, Brazil; ^10^Electrical and Computer Engineering Graduate Program, Federal University of Rio Grande do Norte, Natal, Brazil; ^11^Department of Social Sciences and Management, Universidade Aberta, Lisbon, Portugal; ^12^Department of Life Sciences, Centre for Functional Ecology—Science for People and the Planet (CFE), Associate Laboratory TERRA, University of Coimbra, Coimbra, Portugal

**Keywords:** interculturality, continuing health education, MOOCs, virtual learning environment (VLE), technological mediation, primary health care (PHC)

## 1 Introduction

Interculturality is increasingly gaining conceptual and operational relevance, especially in public health, since the cultural diversity of population groups generates myriad needs and demands for health services. In this scenario, technology-mediated Massive Open Online Courses (MOOCs) have become innovative strategies for health training from an intercultural perspective (1, 2). In Brazil, this is exemplified by the reinforcement of comprehensive Indigenous health care initiatives under primary health care. Prison health is also part of this spectrum of measures, as seen in the National Policy for Comprehensive Healthcare for People Deprived of Liberty. Hence, the prison population is a significant example of cultural and social diversity (3–7).

Consideration of interculturality in the articulation of these initiatives has proven essential for health protection, promotion, and recovery across each community. It favors approaches attuned to traditional knowledge systems and practices specific to culturally diverse groups. Utilizing MOOCs in health, aligned with intercultural principles, enables professionals to be better equipped to work in culturally diverse contexts (8). MOOCs with an intercultural focus have emerged as strategic tools to broaden access to health training and facilitate intercultural practices in health professionals' practice (9, 10).

Interculturality is defined as a “process of interaction and exchange between different cultures aimed at mutual understanding, valuing diversity, and building peaceful and harmonious coexistence” (11). For Wang et al. (12), embracing interculturality in health care requires an understanding of the cultural norms, beliefs, and values adopted by groups in their ancestral health practices. This is essential for delivering universal and equitable health services and practices that respect people's cultural realities, traditions, and cosmovisions. Cunha et al. (11) found that by incorporating intercultural principles, health-related MOOCs help train professionals who are more culturally aware and sensitive to sociocultural diversity.

In this context, the continuing education of health professionals serves as a means to understand the nexus between interculturality and health. Canty (13) highlights interculturality as a central concept to be addressed in MOOCs, which are widely used in the training of health professionals. The author emphasizes that MOOCs go beyond delivering technical knowledge to health professionals; by incorporating interculturality, they facilitate cultural exchange and foster more humanized, comprehensive, and inclusive health care practices.

Health training, although traditionally centered on the technical and scientific updating of professionals, has been redefined in the Brazilian context as a key component of continuing health education (CHE). This shift recognizes that professional development must be intrinsically linked to social needs and to the demands faced by the Brazilian National Health System (SUS) (14, 15). Technology-mediated CHE, particularly through Virtual Learning Environments (VLEs) and the adoption of MOOCs, has been consolidated as a fundamental strategy for the ongoing qualification of health professionals operating nationwide within the SUS (16). This approach promotes and strengthens the connection between health professionals and the population at both individual and collective levels, facilitating communication and supporting the development and adherence to health-promoting programs and practices (7, 12, 17). Therefore, CHE mediated by technology—such as the delivery of MOOCs and Open Educational Resources (OERs) in virtual learning environments—enhances the quality of healthcare services by overcoming geographical, cultural, and linguistic barriers to health promotion (18–20).

One of the main global platforms for providing health-related MOOCs and OERs is the Virtual Learning Environment of the Brazilian Health System (AVASUS). AVASUS is notable for promoting large-scale education with an emphasis on emerging topics relevant to public health (4, 21). Such a platform from Brazil's Ministry of Health was developed by the Laboratory for Technological Innovation in Health (LAIS) at the Federal University of Rio Grande do Norte (UFRN) through technical and scientific cooperation (7). AVASUS is currently the third-largest MOOC platform in the world, with over 3.3 million enrollments, 1.3 million users, and more than 450 open and free courses (16), totalling over 10,000 hours of content. Given its scientific, social, and national significance, AVASUS has been the subject of research, analysis, and evaluation concerning the impact of the massive MOOC and OER delivery within Brazil's SUS.

In light of the above, this study aimed to structure and provide a database to enable an in-depth analysis of the relationship between interculturality and health education within primary health care. The compiled data includes demographic and geographical information on participants in 16 MOOCs offered via AVASUS, comprising 99,080 enrollees from various regions of Brazil and abroad. By compiling this information, this study aims to provide empirical support for research investigating how cultural and territorial factors influence access to, participation in, and ownership of health education content. Thus, it contributes to understanding the multiple sociocultural contexts inherent to professional training, as well as to identifying pedagogical strategies that are more sensitive to diversity and effective in promoting inclusive, comprehensive, and humanized health practices.

## 2 Materials and methods

### 2.1 Data acquisition

The proposal for this study was developed using data extracted from AVASUS, covering 16 courses in the field of primary health care. Data on 99,080 enrollments and attributes spanning from December 3, 2021, to February 27, 2025, were analyzed. Emphasis was placed on elements of interculturality identified, namely: Primary health care as a space for the encounter of diverse forms of knowledge; diversity of regional and socio-cultural contexts of course participants; and valuing data that demonstrate training practices sensitive to local characteristics and specificities.

The attributes, which the set of data on the course participants—except for the unique identifier of the instances (ID), a unique code assigned to each record—, cover sociodemographic, professional, and enrollment data. This includes courses attended, enrollment and completion dates, evaluation, and comments on the course, gender, year of birth, location (city, state, and region of residence), occupation, and health facility of work. The attributes also included content that promotes dialogue of knowledge, appreciation of diverse cultural practices, and inclusion of approaches aimed at equity and diversity in health care.

The database combines quantitative and qualitative variables, allowing for broad and integrated analysis; multivalued data, such as participants operating in multiple fields or having taken several courses; and attributes with missing values, common in large databases. These elements, far from representing limitations, offer opportunities to understand patterns of participation, engagement, and the sociocultural contexts that influence health education.

The study incorporated additional information from external sources to enrich the analysis and ensure methodological rigor. Three data sources were used: (i) the National Register of Health Facilities (CNES), to identify the types and distribution of health services where the participants work and observe regional variations and specific work contexts; (ii) the Brazilian Classification of Occupations (CBO), to characterize participants' professional profiles, facilitating the analysis of how different health functions relate to culturally sensitive practices; and (iii) Brazilian Institute of Geography and Statistics (IBGE), which provides socio-economic and demographic data by region, essential for understanding participants cultural and territorial backgrounds.

By integrating these sources, it was possible to contextualize the educational data with information on the territories, populations, and functions performed, allowing a richer reading of the intercultural dynamics in health education. All the data have been anonymized, organized, and made publicly available in the Zenodo repository (https://doi.org/10.5281/zenodo.15575008). Of note, this study did not involve experimentation with human beings and is therefore not subject to the requirement of ethical approval, under the guidelines established by Resolutions 510/2016 (22) and 674/2022 of CEP/CONEP (23) in Brazil.

### 2.2 Data processing

The original AVASUS data was submitted to a pipeline structured in four stages, developed and implemented in the Python programming language, using widely adopted libraries. The process consisted of the following stages: (i) data quality assessment; (ii) data integration and standardization; (iii) feature extraction; and (iv) feature selection. All stages were conducted in an environment configured with Python 3.10.12, using specialized libraries such as NumPy, Pandas, Matplotlib, Seaborn, and Enelvo, ensuring greater efficiency and reproducibility in data processing (7, 24, 25).

In (i) data quality assessment, the dataset was thoroughly inspected to identify instances containing missing values, inconsistencies, or noise. In (ii) data integration and standardization, null values were replaced, and data formats—such as categories and dates—were standardized. Participants without a formal professional affiliation or a valid CBO code were labeled “individuals with no formal affiliation.” For attributes related to gender, it was necessary to standardize the nomenclatures using the categories: “Female,” “Male,” and “Not reported.” In (iii) feature extraction, attributes were created referring to the region of residence and the descriptive classification of the students' professions. Using the codes from the CBO database (26), a decoding procedure was carried out, allowing the names of the occupations to be integrated into the dataset. To minimize the dispersion of synonymous occupations, a treatment based on regular expressions was applied. For example, variations in descriptions within the field of medicine (such as different medical specialties) were grouped under a single category named “Physician.” Finally, in (iv) feature selection, key attributes for the study's descriptive analysis were defined. A thorough review ensured data consistency, coherence, and anonymization, preparing it for public availability.

## 3 Descriptive analysis

The data reflect a significant panorama of educational participation in the context analyzed, considering interculturality as a structuring dimension in professional health training. The total of 99,080 enrollments in the 16 AVASUS courses indicates increased access to educational opportunities. Despite a standardized rate of 48.79 enrollments per 100,000 population, this representation remains limited considering Brazil's sociodemographic diversity. This underscores the need for strategies that promote greater inclusion and accessibility, respecting the cultural, linguistic, and territorial specificities of diverse social groups.

The ratio between the total number of students (71,961) and the total number of enrollments reflects sustained engagement, as many participants enroll in multiple courses, indicating continued interest in learning (Table 1). This trend may be influenced by cultural factors rooted in the local context, such as the pursuit of training aligned with regional health needs or the promotion of flexible teaching methods that accommodate diverse socioeconomic realities.

**Table 1:** Analysis by course.

**ID**	**Course**	**Enrollments (%)**	**Completed (%)**	**Evaluations**	**Rating ($\bar{x}$)**
1	Introduction to integrative and complementary health practices: aromatherapy	23,709 (23.93%)	5,802 (24.47%)	4,348	4.96
2	National immunization program and primary health care: potential of the family health strategy in rescuing the success story of immunization in Brazil – UPDATED	11,168 (11.27%)	6,805 (60.93%)	6,083	4.97
3	Notions of epidemiological surveillance: looking at the specificities of syphilis; HIV/AIDS; viral hepatitis; tuberculosis – UPDATED	9,318 (9.4%)	4,897 (52.55%)	4,194	4.97
4	Health care for people with disabilities	8,742 (8.82%)	6,189 (70.8%)	5,141	4.96
5	Principles of epidemiological surveillance	8,085 (8.16%)	5,319 (65.79%)	3,669	4.97
6	Training on strategies for the use and distribution of rapid tests for HIV, syphilis, and hepatitis B and C in Brazil – UPDATED	5,928 (5.98%)	4,007 (67.59%)	3,439	4.97
7	Introduction to integrative and complementary health practices: yoga	5,713 (5.77%)	1,429 (25.01%)	1,036	4.94
8	Urgency and emergency in primary health care	5,408 (5.46%)	2,986 (55.21%)	2,480	4.97
9	Urodynamic testing in gynecological clinical practice	4,940 (4.99%)	3,013 (60.99%)	2,442	4.95
10	Access and welcoming of key populations for HIV/AIDS in health services – UPDATED	4,504 (4.55%)	3,334 (74.02%)	2,770	4.98
11	Improving the provision of post-exposure prophylaxis (PEP) for HIV, sexually transmitted infections, and viral hepatitis	4,451 (4.49%)	2,897 (65.09%)	2,466	4.97
12	Update of the main recommendations of the clinical protocol and therapeutic guidelines for hepatitis B and co-infections	2,688 (2.71%)	1,840 (68.45%)	1,565	4.97
13	Approach to smoking patients in primary health care	1,459 (1.47%)	902 (61.82%)	772	4.96
14	The right to health in primary care	1,304 (1.32%)	795 (60.97%)	664	4.96
15	Person-centered consultation and care approach	883 (0.89%)	462 (52.32%)	404	4.97
16	SINAN – notifiable health conditions	780 (0.79%)	257 (32.95%)	220	4.88

### 3.1 Learner demographics

**Figure 1:**
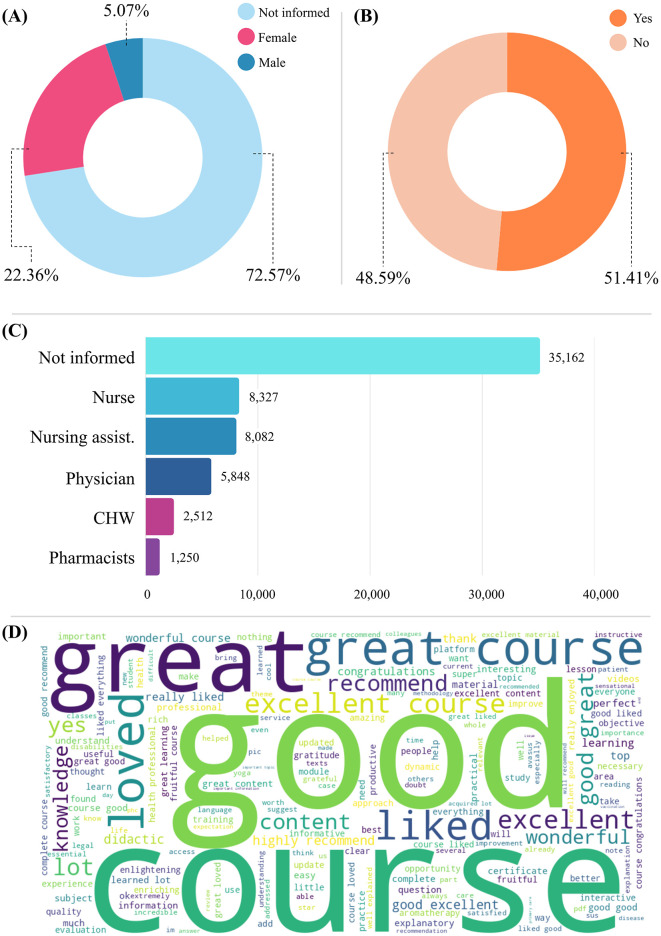
Data analysis. **(A)** Gender distribution among course participants. **(B)** Eligibility for certificate issuance. **(C)** Ranking of professions (unreported plus top five professions). **(D)** Word cloud illustrating participants' evaluations of the courses.

Analysis of the variable gender by course participants revealed that 72.57% of records lack this information, limiting insights into participation dynamics. However, among the available data, female participants predominated (22.36%) compared to males (5.07%) (Figure 1A). This difference may reflect cultural and social patterns related to the division of labor in healthcare, as well as barriers to access for certain groups. It is worth noting that women account for the majority of the workforce in the health sector, according to data from the SUS national gender equity program (27). The lack of more detailed information on gender identity and ethnic-racial belonging indicates the need for more inclusive approaches to data collection and analysis, allowing for a more representative view of the different communities involved in professional health training.

Among the most represented professions, nursing and medical workers predominate, including 8,327 nurses (11.57%), 8,082 nursing aides or assistants (11.23%), and 5,048 physicians (8.13%). In addition, there are 2,512 community health workers (3.49%) and 1,250 pharmacists (1.74%). However, 10,780 (14.98%) of the course participants reported working in other professions, and 35,162 (48.86%) did not state their profession. This predominance shows that the platform is more widely used by professionals directly involved in clinical and care work (Figure 1C).

**Figure 2:**
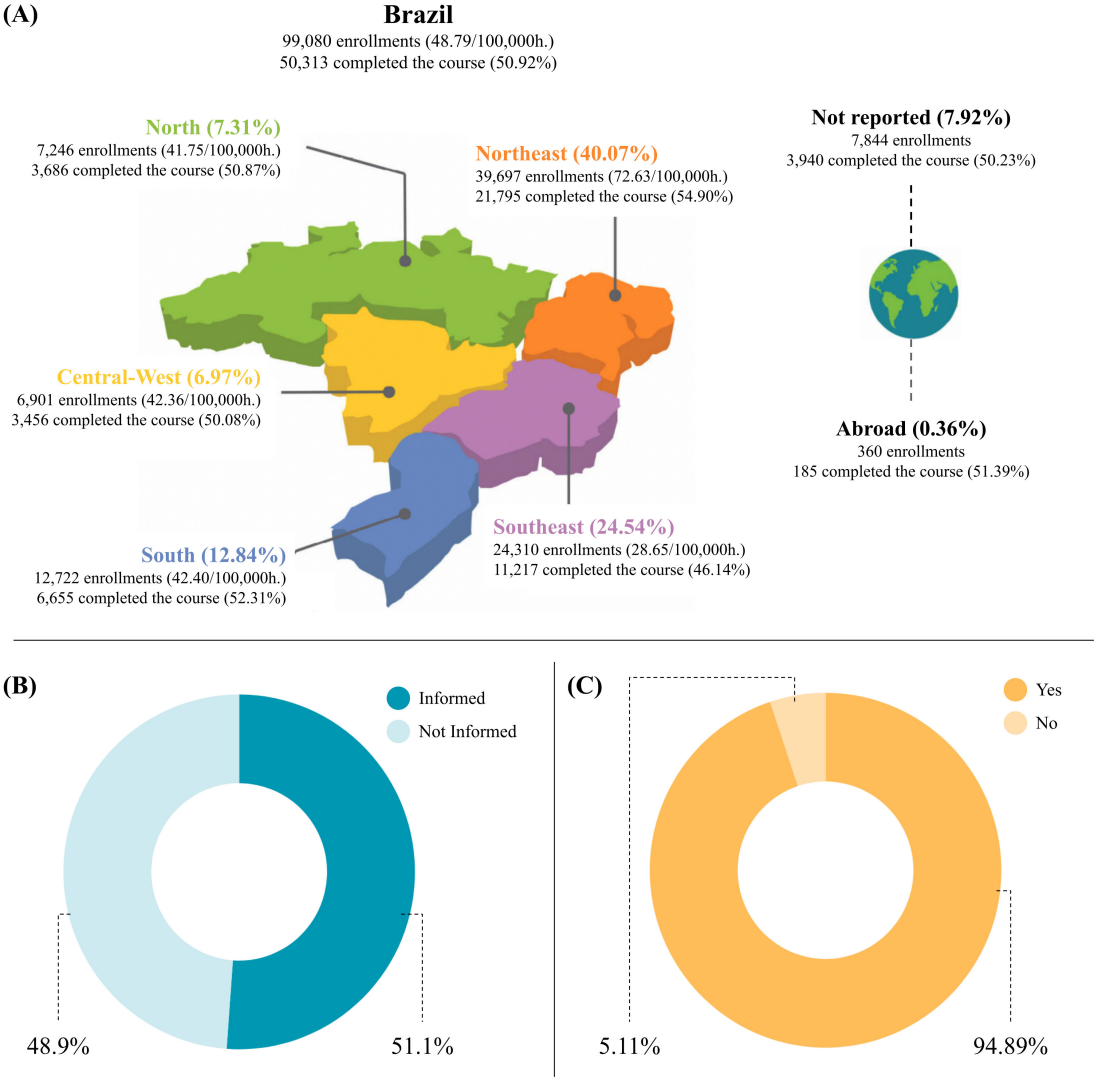
Course participants by Brazilian region and health facilities. **(A)** Participants enrolled by region. **(B)** Participants by health facilities. **(C)** Participants actively providing services in health facilities.

As for the course enrollments by region, the Northeast recorded the highest engagement rate, corresponding to 40.07% of total enrollments. The Southeast region accounted for 24.54% of enrollments, the South for 12.84%, the North for 7.31%, and the Midwest region for 6.97%. International students accounted for 0.36% of enrollments, while 7.92% did not report their region or country of origin (Figure 2A).

The analysis of the variable course participants by health facility revealed that, out of 71,961 course participants enrolled in at least one of the 16 courses, 36,799 (51.14%) informed the health facility where they work, showing a direct connection with the territory and care practices in different contexts (Figure 2B). Of these, 34,918 (94.89%) participants operate in health facilities linked to Brazil's National Health System (SUS). This finding reveals the prominent role of the courses in the humanistic training of professionals within realities marked by socio-cultural diversity (Figure 2C).

### 3.2 Completion behavior

As for the right to certification variable, the figures show that approximately 50% of the total number of students enrolled in the 16 courses completed 100% of the course content. As a result, they were eligible to receive a course completion certificate. Of the total of 99,080 course participants, 50,934 (50.92%) completed 100% of the course and obtained the right to a certificate. In contrast, 48,146 (49.08%) did not finish the course and consequently did not receive the right to obtain the certificate (Figure 1B).

### 3.3 Course quality

The data shows strong overall course performance, notably high average ratings—nearly all courses exceeded 4.9 out of 5—, reflecting positive participant perception. Course 1, despite accumulating the highest number of enrollments, has a relatively low completion rate (24.47%), suggesting possible disengagement or difficulties during the process. In contrast, Course 10 (“Access and Welcoming of Key Populations for HIV/AIDS in Health Services” and Course 4 (“Health Care for People with Disabilities”) attained favorable completion rates (over 70%) and good evaluations, indicating interest and effectiveness. Updated courses also performed well in terms of completions and evaluations, indicating that the relevance and timeliness of content are key to participant engagement.

The word cloud with the course participants' evaluations, with 15,333 comments left by the course participants, corresponding to 30.47% of the completed enrollments, offers key insights into the educational experience on AVASUS (Figure 1D). Predominant terms such as “great,” “excellent,” “good,” “wonderful,” “I liked it,” and “I loved it” denote participants' satisfaction with the content and its applicability. The challenges reported mainly concerned technical difficulties or suggestions for course improvement.

As for the course enrollments by region, the Northeast recorded the highest engagement rate, corresponding to 40.07% of total enrollments. The Southeast region accounted for 24.54% of enrollments, the South for 12.84%, the North for 7.31%, and the Midwest region for 6.97%. International students accounted for 0.36% of enrollments, while 7.92% did not report their region or country of origin (Figure 2A).

The analysis of the variable course participants by health facility revealed that, out of 71,961 course participants enrolled in at least one of the 16 courses, 36,799 (51.14%) informed the health facility where they work, showing a direct connection with the territory and care practices in different contexts (Figure 2B). Of these, 34,918 (94.89%) participants operate in health facilities linked to Brazil's National Health System (SUS). This reveals the strong presence of the courses in the human formation of professionals within realities marked by socio-cultural diversity (Figure 2C).

## 4 Concluding remarks

The data presented have the potential to substantially contribute to studies on interculturality in the context of technology-mediated continuing health education. It recognizes that health professionals and course participants operate in settings shaped by traditional knowledge, local health practices, and different worldviews. Moreover, the sociocultural diversity embedded in Brazil's National Health System (SUS) underscores the need for educational approaches that integrate interculturality into the training of health professionals.

Although the database includes relevant sociographic information—such as age, gender, nationality, and place of birth—, variables that are more sensitive to cultural and intercultural dimensions are still needed, such as ethnic group, religion, belief system, spoken and mother tongues, family situation, socioeconomic level, previous education, and experiences in varied sociocultural contexts. The absence of such data limits a more in-depth understanding of how these aspects influence engagement with courses and professional practice. Acknowledging this gap, we highlight that these aspects will be addressed in future research to broaden the analysis of the impacts of intercultural education on public health.

Finally, strengthening CHE has proven fundamental for building more inclusive and equitable health systems that are sensitive to sociocultural diversity. By preparing professionals to recognize and engage with traditional knowledge, languages, beliefs, and local practices, CHE contributes to improving access, the quality of care, and the relationship between health services and communities. In this sense, the impacts of intercultural dynamics on education go beyond instrumental training, fostering social transformations that reverberate in how health is conceived, practiced, and socially guaranteed.

## Data Availability

The datasets presented in this study can be found in online repositories. The names of the repository/repositories and accession number(s) can be found below: https://doi.org/10.5281/zenodo.15575008.
